# Molecular mechanism for sphingosine-induced *Pseudomonas* ceramidase expression through the transcriptional regulator SphR

**DOI:** 10.1038/srep38797

**Published:** 2016-12-12

**Authors:** Nozomu Okino, Makoto Ito

**Affiliations:** 1 The Department of Bioscience and Biotechnology, Graduate School of Bioresource and Bioenvironmental Sciences, Kyushu University, 6-10-1 Hakozaki, Higashi-ku, Fukuoka 812-8581, Japan

## Abstract

*Pseudomonas aeruginosa,* an opportunistic, but serious multidrug-resistant pathogen, secretes a ceramidase capable of cleaving the *N*-acyl linkage of ceramide to generate fatty acids and sphingosine. We previously reported that the secretion of *P. aeruginosa* ceramidase was induced by host-derived sphingolipids, through which phospholipase C-induced hemolysis was significantly enhanced. We herein investigated the gene(s) regulating sphingolipid-induced ceramidase expression and identified SphR, which encodes a putative AraC family transcriptional regulator. Disruption of the *sphR* gene in *P. aeruginosa* markedly decreased the sphingomyelin-induced secretion of ceramidase, reduced hemolytic activity, and resulted in the loss of sphingomyelin-induced ceramidase expression. A microarray analysis confirmed that sphingomyelin significantly induced ceramidase expression in *P. aeruginosa*. Furthermore, an electrophoretic mobility shift assay revealed that SphR specifically bound free sphingoid bases such as sphingosine, dihydrosphingosine, and phytosphingosine, but not sphingomyelin or ceramide. A β-galactosidase-assisted promoter assay showed that sphingosine activated ceramidase expression through SphR at a concentration of 100 nM. Collectively, these results demonstrated that sphingosine induces the secretion of ceramidase by promoting the mRNA expression of ceramidase through SphR, thereby enhancing hemolytic phospholipase C-induced cytotoxicity. These results facilitate understanding of the physiological role of bacterial ceramidase in host cells.

Sphingolipids are ubiquitous components of eukaryotic cell membranes; however, these sphingoid base-containing lipids are not present in prokaryotes, except for specific bacteria such as *Sphingomonas* and *Sphingobacterium*^1^. In mammals, sphingolipids are well-known lipid mediators that participate in a member of cellular events such as cell growth, differentiation, and apoptosis^2^,^3^. In the sphingolipid-mediated signaling cascade, sphingolipid metabolites exert different biological activities, and, thus, enzymes involved in the sphingolipid metabolic pathway have been intensively studied^4^. Accumulating evidence has indicated that sphingomyelin (SM) is enzymatically converted to ceramide (Cer), sphingosine (Sph), and then Sph 1-phosphate (S1P) by sphingomyelinase (SMase), ceramidase (CDase) and Sph kinase, respectively^2^. S1P is degraded by S1P lyase to form hexadecenal and phosphoethanolamine, which are further metabolized to fatty acids and glycerophospholipids, respectively^5^.

CDase (EC 3.5.1.23) is an amidohydrolase capable of cleaving the *N*-acyl linkage between Sph and the fatty acids of Cer^6^. CDase has been classified into three groups, i.e., acid, neutral, and alkaline CDases depending on optimal pH and primary structures. Acid CDase, composed of α- and β-subunits, is involved in the catabolism of Cer in lysosomes^7^. Neutral CDase, a single polypeptide enzyme, is localized at the plasma membrane as a type II integral membrane protein or is present as a soluble form^8^. Alkaline CDase, the smallest polypeptide among the three CDases, is localized at the ER or Golgi apparatus, at which they participate in controlling intracellular Cer levels^9^. The three CDases are not related to each other based on their primary structures and may have evolved from different ancestor genes. The distribution of the three CDases is somewhat different; however, mammals possess the three types of CDase, i.e., acid CDase is found in vertebrates only, whereas alkaline CDase has been detected in yeasts and mammals, but not in prokaryotes. On the other hand, the genetic information on neutral CDase is conserved from bacteria to humans. We previously identified the neutral CDase in *Pseudomonas aeruginosa* (formerly designated as alkaline CDase due to an optimum pH at 8.5, but was re-classified into neutral CDase after real alkaline CDases showing an optimum pH at 9–10 were found) and cloned its gene^8^,^10^,^11^. Furthermore, we elucidated the crystal structure of *P. aeruginosa* CDase, and demonstrated that it cleaved the *N*-acyl linkage of Cer using a similar mechanism to a zinc-dependent carboxypeptidase^12^.

The Gram-negative bacterium *P. aeruginosa* is a widespread environmental bacillus that causes a wide range of severe opportunistic infections in individuals with weakened immune systems^13^,^14^. However, healthy individuals may also develop mild illnesses caused by this bacterium. Furthermore, an increase in multidrug-resistant strains of *P. aeruginosa* is a serious issue for hospital-acquired infections. *P. aeruginosa* is known to produce multiple virulence factors including proteases, lipases, phospholipases, and exotoxins. These virulence factors play important roles when *P. aeruginosa* invades host cells and causes severe cellular damage^15^,^16^. Among these virulence factors, hemolytic phospholipase C (PlcH) hydrolyzes phosphatidylcholine (PC) and SM to generate diacylglycerol (DG) and Cer, respectively, and phosphorylcholine. Since PC and SM are ubiquitous lipid components in mammalian membranes, PlcH lyses human and sheep erythrocytes^17^,^18^ and causes severe damage to host cells^19^,^20^. We also demonstrated that PlcH-induced hemolysis was significantly attenuated in CDase-deficient mutants of *P. aeruginosa*, indicating that CDase is also a virulence factor of *P. aeruginosa* that enhances the cytotoxicity of PlcH^21^.

*P. aeruginosa* possesses metabolic pathways for various lipids in order to utilize them as nutrients, and, thus, this microbe has the ability to survive under physiologically severe conditions^22^,^23^,^24^. For example, PC is initially hydrolyzed to phosphorylcholine and DG and is then further catabolized to phosphate, choline, glycerol, and fatty acids, which are utilized by *P. aeruginosa* as sources of phosphorus, nitrogen, and carbon^23^,^25^.

Although *P. aeruginosa* possesses PlcH and neutral CDase (CerN, neutral CDase of *P. aeruginosa* is registered as CerN in the *P. aeruginosa* genome database), this pathogen does not possess sphingolipids such as SM, Cer, and Sph. PlcH and CerN were found to be secreted into the culture medium of *P. aeruginosa* when SM was added^21^. The expression of PlcH in *P. aeruginosa* is transcriptionally controlled by the concentration of phosphorus ions; however, the mechanism by which sphingolipids induce the expression of CerN currently remain unclear. In this study, we identified the transcriptional regulator SphR, which mediates the gene expression of CerN in response to sphingolipids, and intensively investigated the specificity of SphR for sphingolipids. It is important to note that the specificity of SphR toward sphingolipids is strict, namely, it responds to free sphingoid bases such as sphingenine, sphinganine (dihydro-Sph), and 4-hydroxysphinganine (phyto-Sph), but not to SM, Cer, 1-deoxy-Sph, or S1P.

This study provides the molecular basis for understanding the mechanisms responsible for the transcriptional regulation of CerN in bacteria that do not possess sphingolipids and facilitates understanding on how pathogens incorporate sphingolipids as a nutrient to survive under severe conditions including the host environment.

## Results

### Identification of gene(s) involved in sphingolipid-mediated CerN induction in *P. aeruginosa*

**Figure 1:**
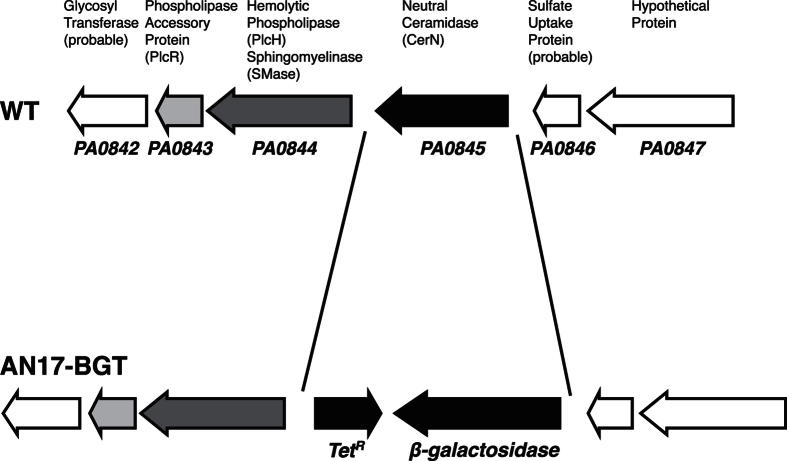
Genomic organization of PA0842-PA0847 in the wild type (WT) and mutant (AN17-BGT) of *P. aeruginosa*. Arrows represent open reading frames and indicate their orientations and sizes.

We previously reported that the release of CerN into a culture medium of *P. aeruginosa* was strongly enhanced by the addition of sphingolipids such as SM, Cer, and Sph^21^. In order to elucidate the molecular mechanism of underlying sphingolipid-mediated CerN induction, we adopted a transposome-based gene disruption strategy in the present study. We initially generated a *P. aeruginosa* mutant (AN17-BGT), in which the *cerN* gene was replaced with the *E. coli β-galactosidase* gene (Fig. 1). Using this mutant, the induction of CerN with sphingolipids was easily monitored by β-galactosidase activity, which may be assessed using chromogenic substrates such as pNP-β-galactose. We then introduced EZ-Tn5 <KAN-2> Tnp Transposome into AN17-BGT and selected mutants lacking β-galactosidase activity in the presence of SM. After the screening of 1,700 mutants, we identified *PA5324* as a candidate gene involved in sphingolipid-mediated CerN induction. In the genome database of *P. aeruginosa* PAO1^26^,^27^, PA5324 was assigned as the AraC-type transcriptional regulator, SphR^28^.

### Functions of SphR in hemolysis caused by *P. aeruginosa*

**Figure 2:**
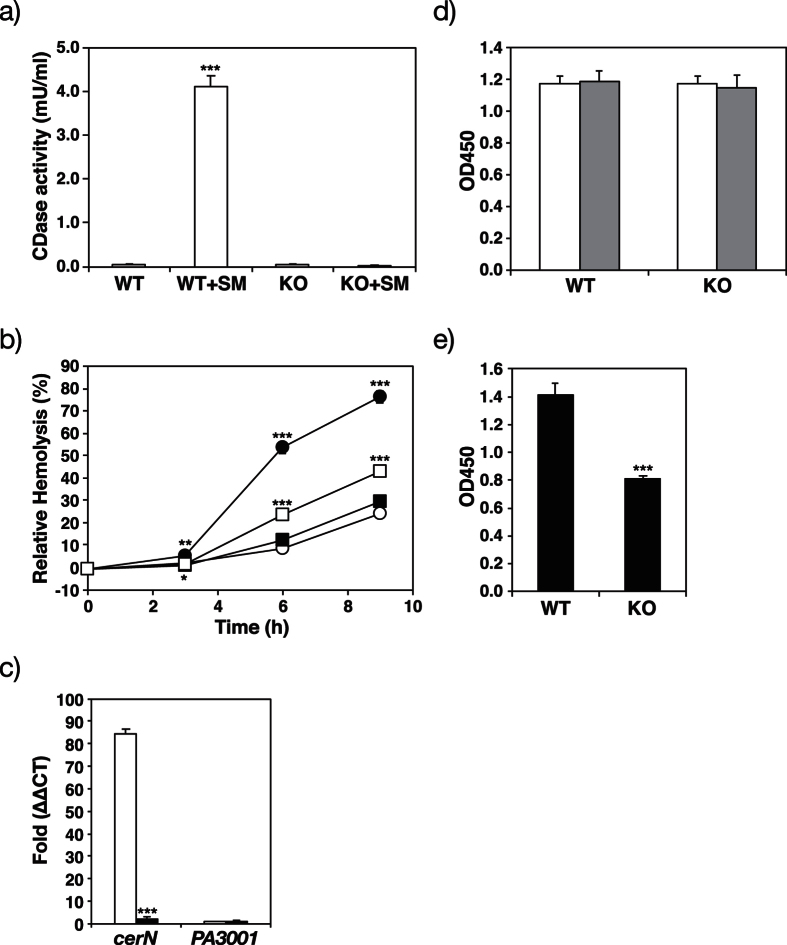
CerN activity, hemolytic activity, *cerN* expression, and cell growth of the wild-type (WT) and SphR-null mutant (KO) of *P. aeruginosa* in the presence or absence of SM. (**a**) CerN activity of the WT and SphR-null mutant of *P. aeruginosa* PAO1. Strains were cultured at 30 °C for 10 h with PY medium in the presence (200 μM) or absence of SM. The CerN activity of the culture supernatant was measured as described in the Methods section. Values are expressed as the mean ± S.D. (n = 3). (**b**) Time course for the hemolysis of sheep erythrocytes by WT, KO, KO complemented with SphR, and the mock transfectant. Hemolytic activity was measured by the method described in the “Methods” section. Values are expressed as the mean ± S.D. (n = 4). ●, WT; ○, KO; □, KO complemented with SphR; ■, mock. Significance is shown as follows: *p < 0.05; **p < 0.01; ***p < 0.001; significantly different from WT for KO or KO complemented with SphR for the mock. (**c**) Expression of *cerN* in the presence or absence of SM. The mRNA levels of *cerN* and *PA3001* were analyzed by real-time RT-PCR. Open and black bars represent the WT and KO, respectively. Values are expressed as the mean ± S.D. (n = 3). Significance is shown as follows: ***p < 0.001 significantly different from WT. (**d** and **e**) Cell growth of the WT and SphR-null mutant of *P. aeruginosa*. (**d**) Strains were cultured at 30 °C for 4 h with 190 μl of PY medium in the absence (open bars) or presence (gray bars) of 200 μM SM. After cultivation, 10 μl of the detection reagent was added to the wells of a 96-well plate and incubated at 30 °C for 2 h. (**e**) Strains were cultured at 30 °C for 20 h with 190 μl of SM synthetic medium (0.05% NH_4_Cl, 0.05% K_2_HPO_4_, 0.5% NaCl, 0.05% TDC, and 0.05% SM). After cultivation, 10 μl of the detection reagent was added to the wells of a 96-well plate and incubated at 30 °C for 1 h. Values are expressed as the mean ± S.D. (n = 4). Significance is shown as follows: ***p < 0.001 significantly different from WT. Statistical analyses were performed by Welch’s t-test.

We generated SphR-deficient mutants of *P. aeruginosa* using the tetracycline resistance (Tc^r^) cassette as described in the “Methods” section. The CerN activities of wild-type *P. aeruginosa* (WT) and SphR-deficient mutants were measured in the presence or absence of SM. As shown in Fig. 2a, CerN activity increased in WT when SM was added to the culture, while its activity was retained at the basal level in SphR-deficient mutants (KO) even in the presence of SM. Similarly, the deletion of SphR in *P. aeruginosa* resulted in hemolytic activity being significantly lower than that in WT, and this was restored in part by the complementation of SphR to the SphR-deficient mutant, indicating that hemolytic activity is also regulated by SphR (Fig. 2b). Since CerN enhances the hemolytic activity of PlcH^21^, these results strongly suggest that SphR plays an important role in hemolysis caused by *P. aeruginosa* through the regulation of CerN.

### Roles of SphR in the transcriptional regulation of CerN

We then examined the roles of SphR in the transcription of the *cerN* gene in *P. aeruginosa* using real-time PCR. The addition of SM resulted in *cerN* transcription levels being more than 80-fold higher in WT than in the absence of SM; however, this sphingolipid-mediated increase in *cerN* transcription was not observed in SphR-deficient mutants (Fig. 2c). The transcription of *PA3001* (housekeeping gene, glyceraldehyde-3-phosphate dehydrogenase) did not increase in WT with the addition of SM. These results indicate that the sphingolipid-mediated induction of CerN occurs at the transcriptional level and is regulated by SphR.

### Effects of the deletion of SphR on *P. aeruginosa* growth

We compared the cell growth of WT and SphR-deficient mutants in nutrition-rich medium (PY medium) and synthetic medium containing SM (SM synthetic medium) as the sole source of carbon. No significant differences were observed in cell growth between WT and SphR-deficient mutants in PY medium in the presence or absence of SM (Fig. 2d). On the other hand, the growth of SphR-deficient mutants was markedly slower than that of WT in SM synthetic medium (Fig. 2e), suggesting that SM is not utilized by SphR-deficient mutants as a source of carbon because CerN was not expressed in *P. aeruginosa* without SphR (Fig. 2a,c).

### DNA microarray analysis

SM is an abundant lipid in the plasma membranes of host cells and may influence the expression of genes involved in SM metabolism in *P. aeruginosa* during the infectious process and colonization. Therefore, we performed a DNA microarray analysis in order to identify the genes induced in *P. aeruginosa* when SM was added to the culture. The mRNA quantity of *P. aeruginosa* was measured using cells cultured in the presence or absence of SM (control). The expression of 94 genes was significantly stronger by more than 1.5-fold (P < 0.05), in the presence of SM than in the control (Table 1). Of those, the expression of 27 genes increased by more than 2-fold, and that of 5 genes by more than 6-fold. SM added to the culture of *P. aeruginosa* may be sequentially converted to phosphorylcholine and Cer by PlcH (SMase), choline and phosphate by phosphatase, and fatty acids and Sph by CerN. Thus, the results of the DNA microarray may reflect gene expression induced not only by SM, but also by SM metabolites such as Cer, Sph, fatty acids, phosphorylcholine, and choline. As expected, the transcript of the *cerN* gene (*PA0845*) showed the greatest increase in *P. aeruginosa* in the presence of SM than in the control (~19-fold, P = 4.91E-6). This result is consistent with those shown in Fig. 2c. The expression of *plcH (PA0844*) and its accessory protein gene (*plcR, PA0843*) also increased in the presence of SM, however, the degree of this increase was lower than *cerN* (2-fold, P < 0.001). The transcriptional level of SphR did not change, whereas those of *PA5325* - *PA5328*, which are adjacent to *sphR (PA5324*) in the *P. aeruginosa* genome, increased by 6~44-fold in the presence of SM; however, the biological relevance of these four genes remains to be clarified. Although transcripts of genes related to sulfur metabolism (*PA0280-PA0283, PA1837-PA1838*, and *PA4442-PA4443*) moderately increased (2~3-fold) in the presence of SM, the mechanism underlying the regulation of these genes by SM is unknown. The expression of *PA5373 (betB*) and *PA5374 (betI*), which form an operon for choline catabolism with *PA5372 (betA*), increased 2-fold in the presence of SM. Furthermore, *PA5375 (betT1*), a putative choline transporter, exhibited a significant increase (1.2-fold, P = 0.0138). The increase observed in the transcription of choline catabolism-related genes (*bet* genes) appeared to be mediated by choline, but not SM^25^. Similarly, the transcription of *Fad* (fatty acid degradation) genes (*PA3014/FaoA/FadB5, PA3013/FaoB/FadA5, PA4994*/probable acyl CoA dehydrogenase, and *PA4995*/probable acyl CoA dehydrogenase) was found to moderately increase in the presence of SM, suggesting that the expression of *Fad* genes in *P. aeruginosa* is regulated by fatty acids released from SM through the corporative reactions of PlcH (SMase) and CerN.

### Lipids inducing *cerN* expression

**Figure 3:**
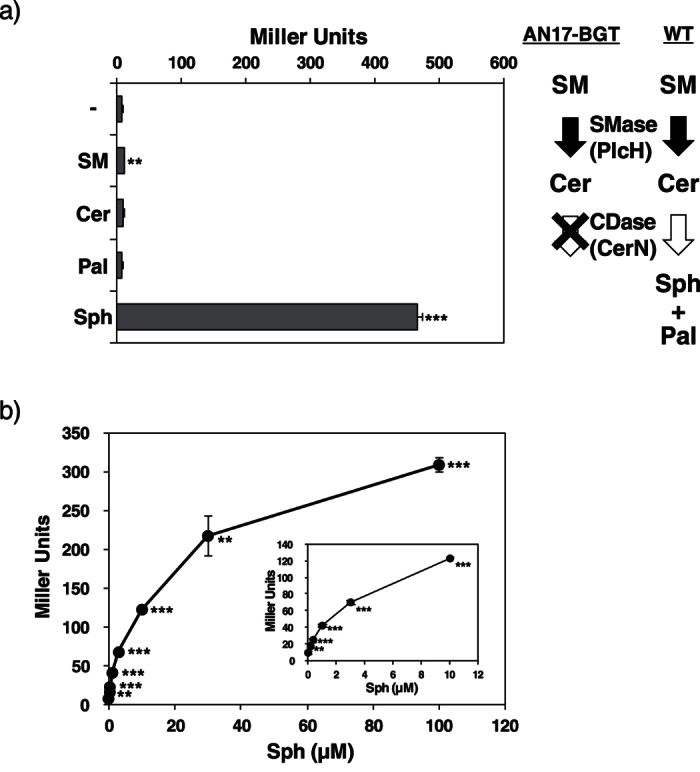
Effects of various lipids on the activation of the *cerN* promoter. (**a**) Effects of SM and its metabolites on the activation of the *cerN* promoter. A 15-μl aliquot of the strain AN17-BGT cell suspension (A600 = 2.0) was inoculated into 1.5 ml of PY medium containing 0.05% sodium taurodeoxycholate in the presence or absence of lipids and cultured at 30 °C for 8 h. After cultivation, promoter activities were measured by the method described in the “Methods” section. -; Control experiment without lipids, SM; sphingomyelin, Cer; ceramide, Pal; palmitic acid, Sph; sphingosine. Values are expressed as the mean ± S.D. (n = 3). **p < 0.01; ***p < 0.001; significantly different from the control experiment without lipids. (**b**) Sph dependence of the *cerN* promoter. A 15-μl aliquot of the AN17-BGT cell suspension (A600 = 2.0) was inoculated into 1.5 ml of PY medium containing 0.05% sodium taurodeoxycholate in the presence of Sph and cultured at 30 °C for 8 h. After cultivation, promoter activities were measured by the method described in the “Methods” section. Values are expressed as the mean ± S.D. (n = 3). **p < 0.01; ***p < 0.001; significantly different from the control experiment without Sph. Statistical analyses were performed by Welch’s t-test.

As previously reported, not only SM, but also its metabolites such as Cer and Sph induce CerN activity in *P. aeruginosa*^21^. However, we found that β-galactosidase activity was only strongly induced when *P. aeruginosa* AN17-BGT was cultured in the presence of Sph, but not SM, Cer, or palmitic acid (Pal) (Fig. 3a). In AN17-BGT, SM may be hydrolyzed to Cer by PlcH, whereas Cer is not converted to Sph because the *cerN* gene was replaced with the *β-galactosidase* gene in this mutant strain. This result suggests that the transcription of the *cerN* gene is induced by Sph, but not SM in *P. aeruginosa* through SphR. In order to elucidate the relationship between the Sph concentrations and extent of the transcription of the *cerN* gene, AN17-BGT was cultured with Sph at various concentrations and β-galactosidase activity was measured. As shown in Fig. 3b, β-galactosidase activity was detected in the presence of no less than 100 nM Sph and activity increased up to 100 μM Sph. These results indicate that the *cerN* gene is transcriptionally activated by Sph in a concentration-dependent manner.

### Analysis of the *cerN* promoter

**Figure 4:**
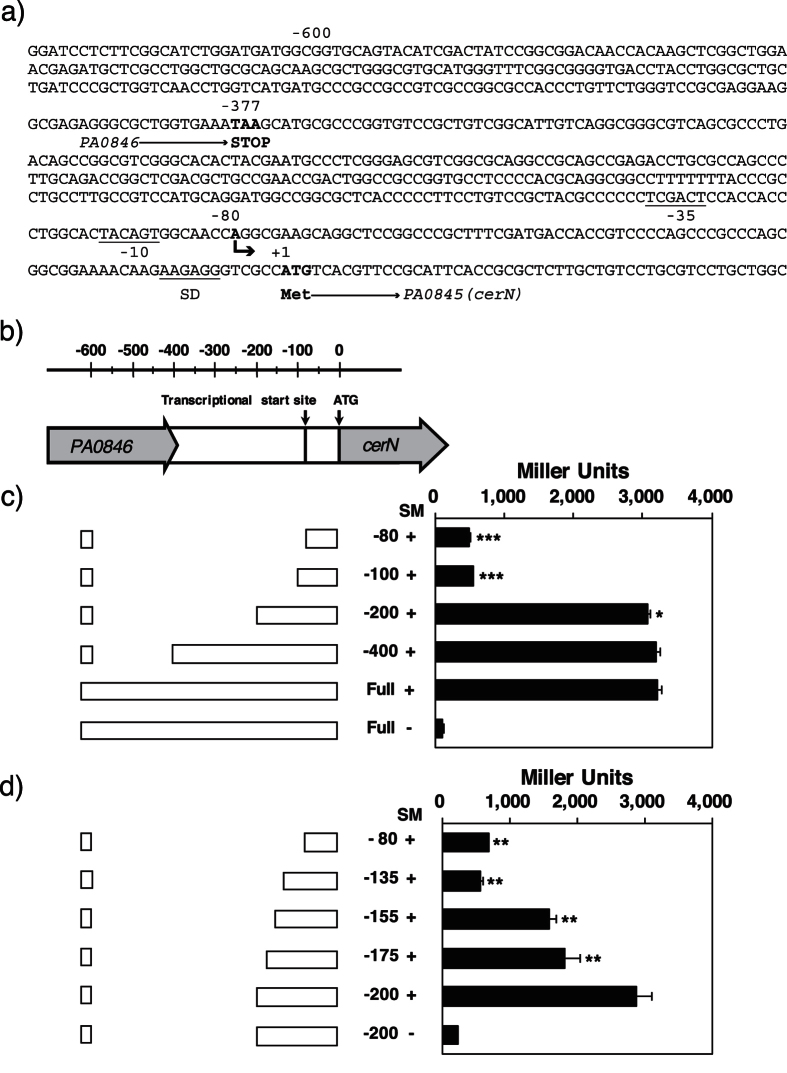
Promoter analysis for the *cerN* gene. (**a**) The transcription initiation site was identified by 5′-RACE. The ATG start codon (*PA0845, CerN*), stop codon (*PA0846*), and *cerN* transcriptional start site (indicated by the bent arrow) are indicated in bold typeface. Underlined nucleotides indicate the putative −35 and −10 promoter sequences and Shine-Dalgarno (SD) sequence. (**b**) A map of the *cerN* promoter region. The ATG start codon and *cerN* transcriptional start site are indicated by arrows. (**c** and **d**) Schematic map of the truncation constructs for the *cerN* promoter and the promoter activity of each construct. A 15-μl aliquot of the cell suspension (A600 = 2.0) was inoculated into 1.5 ml of PY medium containing 0.05% sodium taurodeoxycholate in the presence of 200 μM SM and cultured at 30 °C for 9 h. After cultivation, promoter activities were measured by the method described in the “Methods” section. Promoter activities are expressed in Miller units. +1 denotes the *cerN* ATG start codon. Values are expressed as the mean ± S.D. (n = 3). Significance is shown as follows: *p < 0.05; **p < 0.01; ***p < 0.001; significantly different from Full in the presence of SM for (**c**) and −200 in the presence of SM for (**d**). Statistical analyses were performed by Welch’s t-test.

By using 5′-RACE, we showed that the *cerN* transcription start point was located 80 nucleotides upstream of ATG (Fig. 4a). In order to identify the minimal promoter region required for *cerN* transcription, we generated plasmid constructs containing different sized promoter regions driven by *LacZ. P. aeruginosa* PAO1 was transformed with the constructs (deletion mutants) and the promoter activities of the mutants were evaluated by measuring β-galactosidase activity in the presence of SM. As shown in Fig. 4b and c, β-galactosidase activity was markedly weaker in the mutant possessing 100-bp, but not 200-bp nucleotides from the transcription start point than in the control possessing the putative full promoter sequence, indicating that an integral region for Sph-inducible transcription is present between −200 and −100 bp upstream of the *cerN* gene. Further investigations using several deletion mutants possessing 135- to 200-bp nucleotides identified the critical region in the *cerN* promoter, which was present between −155 and −135 bp upstream of the *cerN* gene (Fig. 4b and d).

**Figure 5:**
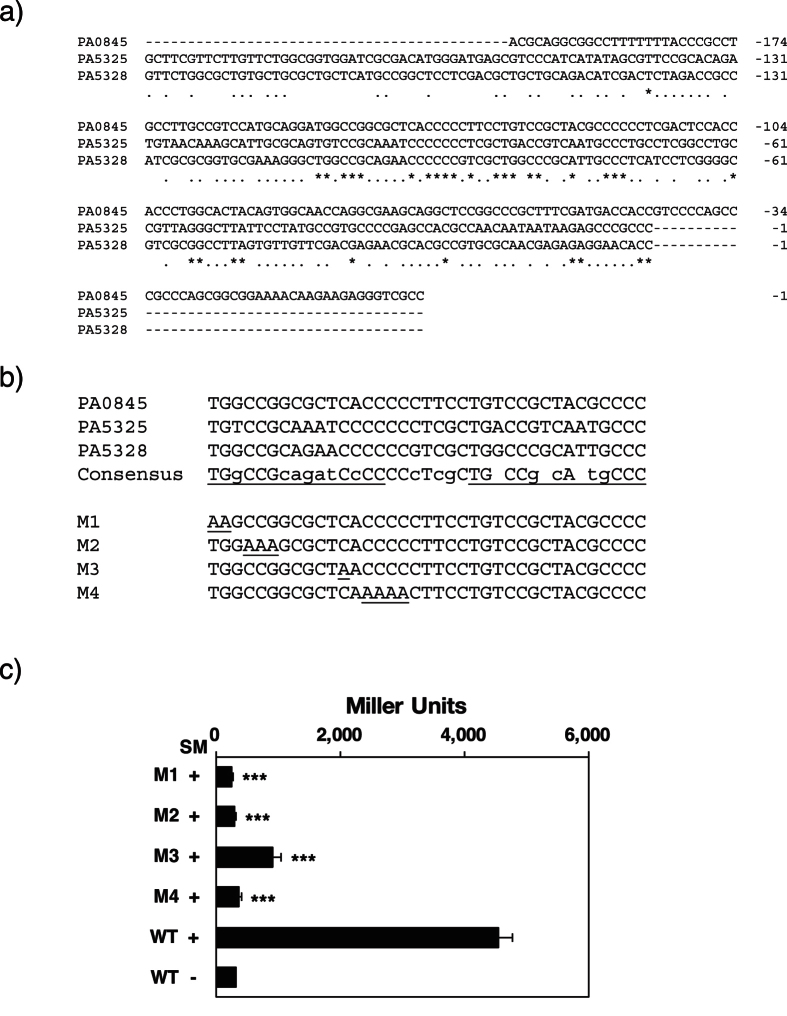
Identification of the SphR-binding sequence in the *cerN* promoter. (**a**) Alignment of the *PA0845 (cerN*) promoter region and 5′-UTR of *PA5325* and *PA5328*. Alignment was performed using the CLUSTAL algorithm^58^. Identical nucleotides in all three sequences are indicated by *asterisks* and identical nucleotides in two sequences are indicated by *dots*. (**b**) Alignment of conserved sequences in the promoter regions of the *PA0845 (cerN*) and 5′-UTR of *PA5325* and *PA5328*. Upper, identical nucleotides in all three sequences and those that are conserved in two sequences are indicated in the consensus sequence by uppercase and lowercase letters, respectively. Putative SphR-binding sequence motifs are underlined. Lower, point mutations (M1~M4) in the conserved region are underlined. (**c**) Promoter activity of each construct. Promoter activities are expressed in Miller units. Values are expressed as the mean ± S.D. (n = 3). Significance is shown as follows: ***p < 0.001; significantly different from WT in the presence of SM. Statistical analyses were performed by Welch’s t-test.

In order to elucidate the nucleotide sequence required for binding to Sph in the *cerN* promoter, the sequence of *PA0845 (cerN*) was aligned with that of the *PA5325* and a *PA5328-PA5326* operon, all of which may be regulated by SphR (Table 1). The conserved nucleotides among these regions were asterisked (Fig. 5a) and the putative consensus sequences were underlined (Fig. 5b). The estimated consensus region appeared to consist of two repeating 15-bp nucleotides separated by a 7-bp spacer when SphR formed a dimer similar to other bacterial transcriptional regulators such as AraC. We generated several mutants (M1–M4), in which a few nucleotides were replaced with A in the conserved sequence and evaluated the promoter activities of these 4 mutants in the presence of SM. As a result, we found that the mutants M1, M2, and M4 completely lost their promoter activities, while the activity of the mutant M3 decreased to one fourth that of WT (Fig. 5c). This result indicates that the conserved sequence is critical for SM-inducible promoter activity.

### Specificity of SphR toward various lipids

**Figure 6:**
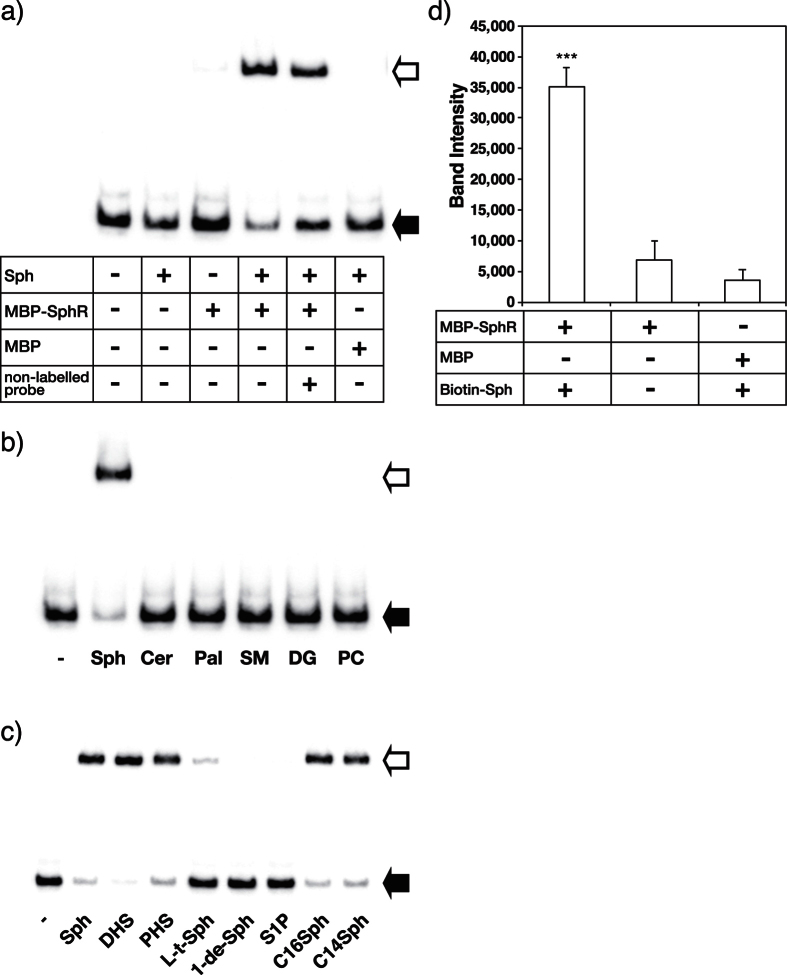
Specificity of MBP-SphR for sphingoid bases. (**a**–**c**) An electrophoretic mobility shift assay (EMSA) showing the DNA-binding activity of MBP-SphR. (**a**) A biotin-labeled probe (0.5 nM) was incubated with MBP-SphR (1.5 μM) or MBP (1.5 μM) as indicated for 30 min, and this was followed by electrophoresis, membrane transfer, and avidin-HRP detection. Ligand specificity was analyzed using various lipids (**b**) and Sph derivatives (**c**). (**b**) -; Control experiment without lipids, Sph; sphingosine, Cer; ceramide, Pal; palmitic acid, SM; sphingomyelin, DG; diacylglycerol, PC; phosphatidylcholine. (**c**) -; Control experiment without lipids, Sph; sphingosine, DHS, dihydrosphingosine, PHS; phytosphingosine, L-t-Sph; L-*threo*-sphingosine, 1-de-Sph, 1-deoxy-sphingosine, S1P; sphingosine-1 phosphate, C16Sph; C16-sphingosine, C14Sph; C14-sphingosine. The unbound probe and shift product are indicated by black and white arrows, respectively. (**d**) Binding of MBP-SphR to biotin-Sph. The binding of MBP-SphR to Sph was assessed using a pull-down assay with biotin-Sph. Values are expressed as the mean ± S.D. (n = 3). Significance is shown as follows: ***p < 0.001; significantly different from the control experiment without biotin-Sph or with MBP. Statistical analyses were performed by Welch’s t-test.

The transcription of the *cerN* gene was induced in *P. aeruginosa* by Sph, but not SM, Cer, or fatty acids (Fig. 3a). We examined whether the Sph-inducible transcriptional regulation of CerN depends on SphR by evaluating the specificity of SphR toward various lipids. Recombinant MBP-fused SphR (MBP-SphR, 84 k) was used in this experiment because free SphR was not soluble under the conditions used. The binding of MBP-SphR to the *cerN* promoter was examined using an electrophoretic mobility shift assay (EMSA), in which the biotin-labeled *cerN* promoter (−200~−81) was used as a probe. As shown in Fig. 6a, MBP-SphR formed a complex with the probe in order to shift up on the gel in the presence of Sph; however, this mobility shift of the probe was not observed in the absence of Sph. The binding of MBP-SphR to the *cerN* promoter attenuated competitively with the addition of the unlabeled probe. No complex was formed with MBP *per se* and the *cerN* promoter. These results indicate that SphR binds to the *cerN* promoter in the presence of Sph. We then evaluated the specificity of SphR to various lipids by EMSA and found that the specificity of SphR was very strict, i.e., SphR bound to Sph, but not SM, Cer, Pal, DG, or PC under the conditions used (Fig. 6b). Among the long-chain bases tested, SphR bound to Sph, dihydroSph, and phytoSph, but not to S1P or 1-deoxy-Sph. Furthermore, SphR recognized the stereoisomers of Sph, i.e., it bound to D-*erythro*-Sph more strongly than L-*threo*-Sph (Fig 6c). These results clearly indicate that SphR recognizes the OH group at C1 and stereochemical configuration at C3 of Sph; however, the double bond between C4 and C5, or hydroxy group at C4 of Sph is not critical for binding to SphR. An acyl chain length of more than 14 in Sph is sufficient for binding to SphR.

In order to examine whether Sph directly binds to purified MBP-SphR, we performed a pull-down assay, in which a complex of MBP-SphR with biotin-labeled Sph was pulled down by avidin-labeled magnetic beads and detected on SDS-PAGE after staining with Coomassie Brilliant Blue. As shown in Fig. 6d, MBP-SphR was only detected in the presence of biotin-labeled Sph and MBP *per se* did not bind to biotin-Sph, indicating that SphR directly binds to Sph.

In conclusion, we demonstrated that Sph specifically binds to SphR, through which the transcription of the *cerN* gene is initiated in *P. aeruginosa*, i.e., the Sph-inducible transcription of CerN entirely depends on SphR as a transcriptional regulator.

## Discussion

In an attempt to elucidate the mechanism underlying the sphingolipid-mediated induction of CerN in *P. aeruginosa*, we examined gene(s) that regulate CerN transcription in response to sphingolipids. As a result, we identified the Sph-specific AraC-type transcriptional regulator, SphR, which is involved in Sph-mediated *cerN* gene expression. AraC-type transcriptional regulators are known to control a number of cellular processes such as carbon metabolism, stress responses, and virulence in Gram-positive and -negative bacteria^29^,^30^. Sixty-one putative AraC-type transcriptional regulators were identified in the *P. aeruginosa* genome when analyzed using a Pfam database^31^. Some AraC-type transcriptional regulators such as VqsM is related to quorum sensing^32^, PchR is involved in pyochelin (siderophore) biosynthesis^33^ and GbdR is involved in phosphorylcholine catabolism^25^,^34^. These transcriptional regulators were previously reported to be important for *P. aeruginosa* infections. AraC-type transcriptional regulators are generally composed of an N-terminal ligand binding domain that is responsible for dimerization and a C-terminal helix-turn-helix (HTH) DNA-binding domain. The binding of a ligand to the N-terminal domain of the transcriptional regulator induces a conformational change, by which it binds to the promoter region of the target gene via a C-terminal domain. We speculated that SphR may be converted to an active form when Sph binds to the N-terminal region, and this active form may then bind to the promoter region of the *cerN* gene via the C-terminal HTH motif, thereby initiating *cerN* mRNA transcription.

LaBauve and Wargo also recently identified SphR in *P. aeruginosa* using a different approach. They performed a DNA microarray in the presence or absence of pulmonary surfactants and found that the expression of several genes including *PA5325* and *PA5328-PA5326* (these three genes are regulated as an operon) increased under the conditions employed. They ascertained that these genes were transcriptionally regulated by Sph in pulmonary surfactants through SphR as a transcriptional regulator^28^. Since Sph is toxic for bacteria and the deletion of SphR was found to decrease the tolerance of *P. aeruginosa* toward Sph, they speculated that *PA5325* (s*phA*) and *PA5328-PA5326 (sphB, C, D*) are genes related to the import and metabolism of Sph in *P. aeruginosa,* respectively.

*P. aeruginosa* synthesizes a small amount of PC (~4% of all phospholipids)^35^,^36^ and appears to possess all the enzymes required to metabolize this phospholipid. Thus, host-derived PC may be utilized by this bacterium as a nutrient for its growth and survival. Host PC is degraded by phospholipase C and DG lipases to phosphocholine, glycerol, and fatty acids in *P. aeruginosa*^23^. Fatty acids, phosphocholine, and glycerol are further metabolized by the β-oxidation pathway, Bet pathway, and Glp pathway in the bacterium, respectively. Son *et al*. found that these genes, which are involved in PC metabolism were up-regulated in *P. aeruginosa* by the addition of PC to the culture^23^. Sun *et al*. also reported that *P. aeruginosa* mutants lacking double or triple genes involved in PC metabolism exhibited a severe growth defect on synthetic medium containing PC as the sole carbon source as well as reduced survival rates in the lungs of mice^37^. These findings indicate that host PC is an integral nutrient for *P. aeruginosa* to infect and survive in the lungs of mice.

SM also appears to be a nutrient for *P. aeruginosa.* SM is a phosphocholine-possessing phospholipid, similar to PC; however, the lipid portion of SM is Cer instead of DG in PC. Host SM has been suggested to be hydrolyzed by two SM hydrolases (PlcB and PlcH) of *P. aeruginosa* in order to generate Cer and phosphocholine^38^. Cer is further decomposed by CerN into Sph and fatty acids. In the present study, we found that not only PlcH and its accessory protein (PlcR), but also the Fad (β-oxidation) and Bet (choline metabolism) genes were up-regulated in *P. aeruginosa* either in the presence of PC or SM. On the other hand, the DG-lipase and Glp (glycerol metabolism) genes were induced by PC, but not SM, while SphA and SphBCD were induced by SM, but not PC in *P. aeruginosa*. These results clearly indicate that SM is metabolized in part by the same pathway as PC; however, the metabolic pathways of Cer (Sph moiety) and DG (glycerol moiety) are completely different from each other in *P. aeruginosa* (see Supplementary Fig. S1).

Several genes were up-regulated in *P. aeruginosa* in the presence of SM, but not PC. Of these, *PA0845* is the gene encoding neutral CDase (CerN) and *PA5325 (sphA*) is the gene encoding a homologue of porin, which makes a channel in the plasma membrane as a transporter. *PA5328-PA5326 (sphBCD*), three genes that form an operon, showed homology to cytochrome C oxidase (cbb3-type, subunit III), D-arabinono-1,4-lactone oxidase, and alanine racemase (N-terminal domain), respectively. These proteins may be involved in Sph transport and metabolism in *P. aeruginosa*; however, the physiological functions of these proteins have not yet been elucidated.

CerN may be induced in *P. aeruginosa* in the presence of not only Sph, but also SM or Cer^21^. However, we found that the promoter of *cerN* was activated by Sph, but not by SM or Cer when the strain ANT17-BGT was used instead of *P. aeruginosa* WT (Fig. 3a). This contradiction may be consistently explained, i.e., SM and Cer are converted to Sph by PlcH (SMase) and CerN in *P. aeruginosa* WT, but are not converted to Sph in the strain AN17-BGT because the CerN gene was replaced with the *E. coli β-galactosidase* gene and, thus, SM/Cer was not converted to Sph. On the other hand, the promoter of *cerN* in the strain AN17-BGT was activated by SM when we screened β-galactosidase-deficient strains (Fig. 1). We found that the egg yolk SM used in this experiment was contaminated with Sph (~0.5% of SM), and, thus, this experiment may be regarded as being performed in the presence of Sph. Thereafter, we used Sph-free SM in the experiments described in Figs 3a and 6b.

**Figure 7:**
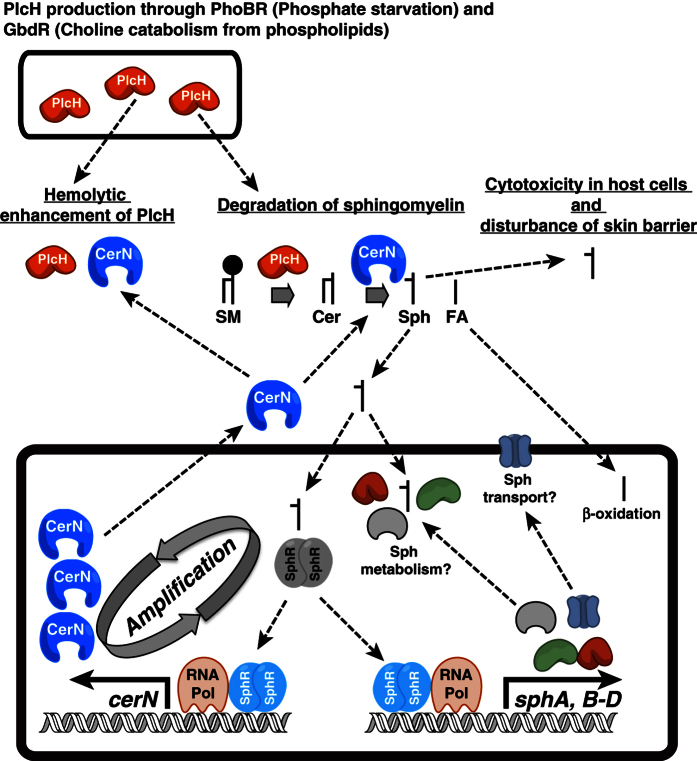
Proposed mechanisms of SphR in *P. aeruginosa*. The model shows Sph-induced *cerN* gene expression through SphR. The generation of Sph is amplified by a positive feedback loop between SphR and CerN. This figure also represents the regulation of intracellular Sph levels in the bacterium by SphR^28^. CerN; neutral CDase, Cer; ceramide, FA; fatty acids, PlcH; hemolytic phospholipase C, RNA Pol; RNA polymerase, SM; sphingomyelin, Sph, sphingosine.

Figure 7 shows a working model for the functions of SphR in *P. aeruginosa*. The presence of a positive feedback loop has been suggested between SphR and CerN, i.e., 1) Host Sph is incorporated into *P. aeruginosa* and induces the expression of CerN through the activation of SphR, 2) CerN is secreted into the host environment, in which host-cell Cer is hydrolyzed to generate Sph, 3) the Sph generated is then incorporated into *P. aeruginosa* and increases CerN expression through the activation of SphR. This positive feedback loop amplifies the generation of Sph in host environments and may exacerbate damage to host cells. In this model, PlcH also plays an important role in the production of Cer from host-derived SM. PlcH is induced by the PhoBR regulon under phosphate starvation conditions and by the GbdR regulon with the catabolism of choline from phospholipids such as PC and SM. On the other hand, SphR regulates intracellular Sph levels in *P. aeruginosa* through the expression of *sphABCD*, which may be involved in the transport and metabolism of Sph^28^. Since Sph exhibited the antibacterial activity toward many bacteria including *P. aeruginosa*^39^,^40^, the regulation of intracellular Sph levels is integral for bacteria exposed to Sph. According to this point of view, the study by Pewzner-Jung *et al*. is of interest. They reported that tracheal and bronchial epithelial Sph levels play important roles in the prevention of *P. aeruginosa* in healthy individuals and Sph levels are reduced in patients with cystic fibrosis due to reductions in acid CDase activity^41^. Collectively, SphR exerts two different physiological functions in *P. aeruginosa,* i.e., attacking host cells through the generation of Sph by the expression of CerN and defending against Sph through a decrease in intracellular Sph levels by the expression of SphABCD.

We found that Sph at a concentration of no less than 100 nM induced the transcription of *cerN* in *P. aeruginosa* (Fig. 3b). This concentration of Sph was markedly lower than its physiological concentration in human serum (~250 nM)^42^, and, thus, we speculate that the *cerN* gene is expressed in *P. aeruginosa* in some host environments. We isolated a CDase-producing bacterium, *P. aeruginosa* AN17, from the skin of patients with atopic dermatitis (AD)^10^ and we demonstrated that the skin of these patients is significantly colonized by CDase-secreting bacteria, including *P. aeruginosa*, compared to that of healthy individuals^43^. The human epidermis contains a certain amount of Sph and dihydroSph^44^, which may induce the expression of *cerN* in *P. aeruginosa*-infected skin through SphR. We found that CerN hydrolyzes Cer in the human skin efficiently in the presence of anionic phospholipids derived from *Staphylococcus aureus*, which is found in atopic skin frequently, in place of detergents^45^. Recently, Oizumi *et al*. demonstrated that Sph produced by the action of CerN is converted to S1P and induces production of inflammatory mediators in human keratinocytes^46^. In addition, exogenous Sph, dihydroSph, and phytoSph are found to be modulators of differentiation and lipid metabolism in keratinocytes^47^,^48^. These observations indicate that CerN disturbs the skin barrier function by decreasing the levels of skin Cer and by increasing the long-chain bases. This study also suggests the important role of CerN as an exacerbating factor in *P. aeruginosa*-infected skin in conditions such as AD and related dermatitis. From another point of view, *P. aeruginosa* is an important opportunistic human pathogen, but is also found in diverse environmental conditions, such as soil and water. Because all eukaryotes and some bacteria possess sphingolipids as constituents of biological membranes, the bacterium may produce CerN through SphR, for decomposing SM or Cer from sphingolipid-producing organisms to obtain fatty acids as a nutrient source.

In order to obtain a better understanding of the relationship between SphR and CerN in bacteria, we performed a blast search using the amino acid sequences of SphR and CerN. Among 56 bacteria with SphR homologues (E-value < e-30), we found that 12 bacteria (including *P. aeruginosa*) had highly homologous sequences of CerN (see Supplemental Fig. S2). Furthermore, their distribution was found in Gram-negative and Gram-positive bacteria, and frequently in the genus *Mycobacterium. Mycobacterium* are well-known pathogens towards humans and animals; however, other bacteria, except for *Collimonas pratensis* and *C. fungivorans*, which have both SphR and CerN, have also been identified as pathogenic bacteria towards humans and animals. Although the functions of SphR homologues currently remain unknown, the distribution of both proteins enables us to verify the importance of SphR in CerN production in pathogenic bacteria.

In the present study, we clarified the mechanism underlying the transcriptional regulation of *cerN* in *P. aeruginosa*, in which SphR functions as an Sph-inducible transcriptional regulator. Furthermore, we present detailed biochemical results on the interaction between Sph and SphR. The results described here may contribute to developing SphR inhibitors that have the potential to become anti-microbial drugs for SphR-possessing pathogens including *P. aeruginosa*. The AN17-BGT strain developed in this study is a useful tool for the high throughput (HST) screening of inhibitors of the interaction between SphR and the *cerN* promoter or SphR and Sph, which is easily performed by detecting β-galactosidase activity using artificial substrates.

## Methods

### Materials

SM, sodium taurodeoxycholate, S1P, palmitic acid, DG, and Triton X-100 were purchased from Sigma-Aldrich Corp. (St. Louis, MO, USA). D-*erythro*-Sph, *N*-palmitoyl-Sph, and dihydro-Sph were purchased from Enzo Life Sciences, Inc. (Farmingdale, NY, USA). Phyto-Sph was purchased from Olbracht Serdary Research Laboratories (Toronto, Canada). Omega-biotinyl D-*erythro*-sphingosine (biotin-Sph), L-*threo*-Sph, and 1-deoxy-Sph were purchased from Avanti Polar Lipids, Inc. (Alabaster, AL, USA). C14-Sph and C16-Sph were purchased from Matreya, LLC (State College, PA, USA). Chlorophenol red-β-D-galactopyranoside (CPRG) was purchased from Roche Diagnostics (Mannheim, Germany). Precoated Silica gel 60 TLC plates were purchased from Merck (Darmstadt, Germany). The pHP45Ωaac vector^49^ was donated by Dr. H. Niki, National Institute of Genetics, Japan. All other reagents were of the highest purity available.

### Bacterial strains and media

A type strain of *P. aeruginosa* PAO1 (JCM14847) was provided by the Japan Collection of Microorganisms, RIKEN BRC, which is participating in the National BioResource Project of MEXT, Japan. *P. aeruginosa* strains were cultivated in PY medium (0.5% polypeptone, 0.1% yeast extract, and 0.5% NaCl, pH 7.2) at 30 °C. *E. coli* strains were grown in Luria- Bertani (LB) medium at 37 °C. Media were supplemented with antibiotics such as ampicillin (100 μg/ml for *E. coli*), carbenicillin (100 μg/ml for *E. coli*), kanamycin (50 μg/ml for *E. coli* and 1 mg/ml for *P. aeruginosa*), gentamycin (20 μg/ml for *E. coli* and 30 μg/ml for *P. aeruginosa*), and tetracycline (15 μg/ml for *E. coli* and 200 μg/ml for *P. aeruginosa*) where necessary.

### Construction of AN17-BGT

All plasmids were constructed using PCR with PrimeSTAR MAX DNA polymerase (Takara Bio, Kusatsu, Japan) and the In-Fusion PCR cloning kit (Takara Bio). A 5-kbp fragment, which contained the 5′-flanking region, open reading frame, and 3′-flanking region of the *cerN* gene, was amplified by PCR using *P. aeruginosa* AN17 genome DNA as a template and the primers PaCD-5k-Infu1 and PaCD-5k-Infu2, and was then cloned into the BamHI site of pGEM-3Zf(+). The resulting plasmid was named pGEM-PaCD5k. In order to replace the *cerN* gene with the *E. coli β-galactosidase* gene, the *cerN* gene was removed by PCR using pGEM-PaCD5k as a template and the primers PaCD-5′UTR and PaCD-3′UTR. The fragment obtained was ligated with the *β-galactosidase* gene amplified from pGEM-LacZ using the primers PaCD-5′UTR-LacZ-5′ and PaCD-3′UTR-LacZ-3′. The resulting plasmid was named pGEM-PaCD5k-LacZ. pGEM-PaCD5k-LacZ was linearized by PCR using the primers PaCD3′UTR-3′U and PaCD3′UTR-5′L to introduce the Tc^r^ cassette into the 3′UTR region of the *cerN* gene in pGEM-PaCD5k-LacZ. The Tc^r^ cassette was amplified by PCR using pBR322 as a template and the primers pBR322-U1-PaCD3′ and pBR322-L1-PaCD3′, and was then ligated to linearized pGEM-PaCD5k-LacZ. The resulting plasmid was named pGEM-PaCD5k-LacZ-Tet. The cassette in pGEM-PaCD5k-LacZ-Tet was amplified by PCR using pGEM-PaCD5k-LacZ-Tet as a template and the primers PaCD Pro-5k-Infu1 and PaCD Pro-5k-Infu2, and was then ligated to the BamHI site of pK19mobsacB^50^. The sequences of the primers used in this study are summarized in Supplementary Table S1. The resulting plasmid was named pK19-PaCD5k-LacZ-Tet. The plasmid, which contains a mutant *cerN* disrupted with the *β-galactosidase* and *Tc*^*r*^ genes, was transferred from the broad host range-mobilizing strain *E. coli* S17-1^51^ to *P. aeruginosa* AN17 by biparental filter matings. Tc^r^ plasmid integrants were selected on NAC medium containing Tc. Tc^r^ colonies were then plated on LB agar containing 5% sucrose to identify strains that had lost the vector-associated sacB gene (resistant to sucrose). Gene replacement was ascertained by PCR of the *cerN* gene. The resulting strain was named AN17-BGT.

### Construction of the AN17-BGT mutant library and screening of β-galactosidase activity-deficient cells

An EZ-Tn5 <R6Kγori/KAN-2> Tnp Transposome kit (Epicentre, Madison, WI, USA) was used to make the AN17-BGT mutant library according to the manufacturer’s instructions. In order to obtain mutants, a 100-μl aliquot of electrocompetent cells was mixed with 1 μl of the EZ-Tn5^TM^ <R6Kγ*ori*/KAN-2> Tnp Transposome^TM^ (Epicentre), and electroporation was then performed with a Gene Pulser II electroporator (Bio-Rad Laboratories, Hercules, CA, USA) in 0.2-cm cuvettes at 2.5 kV, 25 μF, and 200 Ω. Cells were transferred to a 10-ml tube containing 2 ml of PY medium and incubated at 37 °C for 2 h with shaking. Transformants were selected on NAC agar containing 1 mg/ml kanamycin. The colonies obtained were added to the wells of a 96-well microplate containing 100 μl of PY medium. On the next day, a 10-μl aliquot of the culture was transferred to a new 96-well microplate containing 100 μl of PY medium with 200 μM SM from chicken egg yolk and incubated at 25 °C for 1 day. The β-galactosidase activity of each mutant was measured using CPRG. In order to permeabilize cells, 10 μl of 0.1% SDS and 1 drop of CHCl_3_ were added to each well and mixed well. The β-galactosidase assay was started by adding 50 μl of 2x Z-buffer (120 mM Na_2_HPO_4_·7H_2_O, 80 mM NaH_2_PO_4_·H_2_O, 20 mM KCl, 2 mM MgSO_4_·7H_2_O, and 100 mM β-mercaptoethanol) and 10 μl of 1 mM CPRG. β-galactosidase activity was measured using a spectrophotometer at 570 nm.

### Construction of the SphR mutant and complementation strains

The upstream and downstream sequence fragments (1,000 bp) flanking the *sphR* gene were amplified by PCR using AN17 genome DNA as a template and the primers SphR-1000-Infu1 and SphR-1000-Infu2. The amplified product was cloned into the BamHI site of pK19mobsacB and the resulting plasmid was named pK19-SphR. In order to replace the *sphR* gene with the Tc^r^ cassette, the *sphR* gene was removed by PCR using pK19-SphR as a template and the primers SphR-KO-InfuU1 and SphR-KO-InfuL1. The fragment obtained was ligated with the Tc^r^ cassette amplified from pBR322 by using the primers pBR322U1new and pBR322L1331new. The primers used are summarized in Supplementary Table S1. The resulting plasmid was named pK19-SphR-Tet. The plasmid containing a mutant SphR disrupted with the Tc^r^ gene was transferred from the broad host range-mobilizing strain *E. coli* S17-1 to *P. aeruginosa* PAO1 by biparental filter matings. Gene replacement was ascertained by PCR of the *sphR* gene. The resulting strain was named ΔSphR. In order to construct the SphR complementation vector, we first introduce the gentamicin resistance (Gm^r^) cassette into the EcoRI site of pMMB66HE^52^. pMMB66HE was linearized by PCR using the primers pMMB66HE-U1 and pMMB66HE-L1. The Gm^r^ cassette was amplified by PCR using pHP45Ωaac as a template and the primers aacC4-1-Infu1 and aacC4-1-Infu2, and was then ligated to linearized pMMB66HE. The resulting plasmid was named pMMB66HE-Gen. The *sphR* gene was amplified by PCR using pK19-SphR as a template and the primers SphR-U1-Infu1 and SphR-L1-Infu2, and was then ligated to the BamHI site of pMMB66HE-Gen, which was linearized by PCR using pMMB66HE-Gen as a template and the primers pMMB66HE-aacC4-U1 and pMMB66HE-aacC4-L1. The resulting plasmid was named pMMB66HE-Gen-SphR. pMMB66HE-Gen-SphR was transferred from the broad host range-mobilizing strain *E. coli* S17-1 to the strain ΔSphR by biparental filter matings. The primers used are listed in Supplementary Table S1.

### Assay of CDase

CDase (CerN) activity was measured using C12-4-nitrobenzo-2-oxa-1,3-diazole (NBD)-Cer as a substrate as previously described^21^.

### Assay of hemolysis

Fifty microliters of the *P. aeruginosa* cell suspension (A600 = 0.4) in saline was mixed with 50 μl of sheep erythrocytes in saline and incubated at 30 °C for an appropriate time. After being incubated, the reaction mixtures were centrifuged at 1,000 × *g* for 3 min, the supernatant was diluted with distilled water (3 volumes), and the release of hemoglobin was then measured using the Multiskan FC Microplate Photometer (Thermo Fisher Scientific, Inc., Waltham, MA, USA) at 492 nm.

### Assay of cell viability

The cell viabilities of the wild-type and SphR-deficient mutant were evaluated by Microbial Viability Assay Kit-WST (DOJINDO LABORATORIES, Kumamoto, Japan). This colorimetric microbial viability assay is based on the reduction of WST-8 reagent by the reducing ability of viable cells^53^,^54^. Strains were grown at 30 °C overnight on NAC agar. Bacteria were then scraped from the agar plate and suspended in saline to an A550 of 0.125. A 10-μl aliquot of the cell suspension (A550 = 0.125) was inoculated into 180 μl of PY medium containing 0.05% sodium taurodeoxycholate in the presence and absence of 200 μM of SM and then incubated at 30 °C for an appropriate time. After the incubation, cell viability was analyzed by measuring the metabolic activity of cells using Microbial Viability Assay Kit-WST according to the manufacturer’s instructions.

### Real-time PCR

The WT and SphR-null mutant of *P. aeruginosa* PAO1 were cultured in PY medium with and without 200 μM SM at 30 °C. After 8 h, cells were stabilized by RNAlater (Thermo Fisher Scientific) and harvested. Total cellular RNA was isolated from the cells using TRI reagent (Molecular Research Center, Inc., Cincinnati, OH, USA), and the SV Total RNA Isolation System (Promega Corporation, Madison, WI, USA). Reverse transcription was performed using the Prime Script RT reagent kit (Takara Bio) in accordance with the manufacturer’s protocol. Real-time PCR was performed using the SYBR Premix ExTaq kit (Takara Bio) in an Mx3000P QPCR System (Agilent Technologies, Santa Clara, CA, USA). The data obtained were normalized against the expression levels of PA3001, which were not affected by the SM treatment, as shown in the microarray analysis. Relative changes in gene expression were analyzed using the ∆∆CT method^55^. The sequences of the primer pairs are described in Supplementary Table S1.

### Microarray analysis

In microarray experiments, *P. aeruginosa* strain PAO1 was cultured in 2 ml of sterilized PY medium, and incubated at 30 °C for 1 day with vigorous shaking. An aliquot of the cell suspension (OD600 = 0.01) was inoculated into 2 ml of PY medium containing 0.05% sodium taurodeoxycholate and 200 μM of SM, and then shaken at 30 °C for 8 h. Cells were stabilized by RNAlater and harvested. Total cellular RNA was isolated from cells using TRI reagent and the SV Total RNA Isolation System. The integrity of total RNA samples was assessed by the Experion Automated Electrophoresis System (Bio-Rad). Labeled cDNA probes were prepared according to the protocol supplied by the manufacturer (Affymetrix, Santa Clara, CA, USA) and hybridized to a GeneChip *P. aeruginosa* Genome Array (Affymetrix) according to the manufacturer’s instructions. Data analysis was initially performed with Affymetrix Expression Console™ Software. Microarray experiments and data analyses were supported by Cell Innovator Inc. (Fukuoka, Japan). Gene expression changes with significance were identified by Linear Models for Microarray Analysis (limma) package^56^ of Bioconductor software. Fold changes were calculated as the average ratios between the signal in the absence (control) and presence of SM. Genes induced by 1.5-fold or more and a *P* value of <0.05 were considered to be significant. Our results have been uploaded to the Gene Expression Omnibus database (accession number GSE83078).

### Identification of the transcriptional start site using 5′-RACE

*P. aeruginosa* strain PAO1 was cultured in 2 ml of sterilized PY medium, and incubated at 30 °C for 1 day with vigorous shaking. An aliquot of the cell suspension (OD600 = 0.02) was inoculated into 2 ml of PY medium containing 0.05% sodium taurodeoxycholate and 100 μM of SM, and shaken at 30 °C for 8 h. Cells were stabilized by RNAlater and harvested. Total cellular RNA was isolated from cells using TRI reagent and the SV Total RNA Isolation System. Purified total RNA was used for transcription start site identification of the *cerN* gene using the 5′-Full RACE Core Set (Takara Bio) according to the manufacturer’s instructions. First-strand cDNA was synthesized by the gene-specific reverse primer PaCDProRT, and first PCR was performed by first-strand cDNA as the template with the primers PaCDPro-A1 and PaCDProS1. Second PCR was performed using the first PCR reactant as the template with the primers PaCDPro-A2 and PaCDProS2, and the product was cloned into the pGEM T-easy vector (Promega) and sequenced. The primers used are listed in Supplementary Table S1. The first nucleotide following the PaCDProRT sequence was taken as the transcriptional start.

### Promoter analysis

The promoter region of the *cerN* gene, *β-galactosidase* gene, 3′-UTR region of the *cerN* gene, and *Tc*^*r*^ cassette were amplified by PCR using pK19-PaCD5k-LacZ-Tet as a template and the primers PaCDPro2-Infu1 and PaCDPro2-Infu2-1. The amplified product was inserted into the linearized pMMB66EH vector, which was amplified by PCR using pMMB66EH as a template and the primers pMMBdel-U1-BamHI and pMMBdel-L1-BamHI. The recombinant plasmid was designated pMMBProCD2. Point and deletion mutations were introduced in pMMBProCD2 using the PrimeSTAR mutagenesis basal kit (Takara Bio) according to the instructions provided by the manufacturer. The primers used for these constructs are listed in Supplementary Table S1. The resulting plasmids were transferred from the broad host range-mobilizing strain *E. coli* S17-1 to *P. aeruginosa* PAO1 by biparental filter matings. β-Galactosidase activities were measured by the method of Miller^57^ using cells permeabilized with chloroform.

### Expression and purification of MBP-SphR

The *sphR* gene was amplified by PCR using pUC-SphR as a template and the primers SphR-pMAL-Infu1 and SphR-pMAL-Infu2 (see Supplementary Table S1). The amplified product was inserted into EcoRI- and NotI-digested pMAL-c5X (New England Biolabs, Ipswich, MA, USA) to generate MBP-SphR, N-terminal fusion to the maltose-binding protein (MBP). The recombinant plasmid was designated pMAL-SphR. *E. coli* strain BL21(DE3) cells were transformed with pMAL-SphR and grown at 25 °C overnight with shaking in 3 ml of Luria-Bertani medium supplemented with 100 μg/ml of carbenicillin. The culture was then transferred to a 500-ml flask containing 200 ml of Luria-Bertani medium supplemented with 0.2% glucose and 100 μg/ml of carbenicillin incubated at 25 °C with shaking to an OD600 of approximately 0.5. Isopropyl 1-thio-D-galactopyranoside (IPTG) was then added to the culture to a final concentration of 1 mM to induce transcription. After an additional 5-h culture at 25 °C, cells were harvested by centrifugation and suspended in 5 ml TBS containing 0.1% Triton X-100. After sonication for 3 min, cell debris was removed by centrifugation (15,000 × *g* for 30 min). The supernatant was loaded onto a column of amylose resin (NEB), and the column was washed with TBS. MBP-SphR was eluted from the column with TBS containing 10 mM maltose. The fractions containing MBP-SphR were pooled and loaded onto a Superdex 200 HR column (GE Healthcare) equilibrated with 25 mM MES buffer, pH 6.0, containing 100 mM NaCl. Since MBP-SphR has the ability to restore CerN activity in the SphR mutant by a plasmid expressing MBP-SphR fusion (data not shown), we used MBP-SphR in subsequent biochemical experiments.

### Electrophoretic mobility shift assay

A 120-bp DNA fragment containing the −200 to −81 region of the *cerN* promoter was amplified by PCR using pPaCDPro400 as a template and the primers PaCDPro-200U and PaCDPro-81L (see Supplementary Table S1). The 120-bp DNA fragment obtained was labeled with the Biotin 3′ End DNA Labeling Kit (Thermo Fisher Scientific). Biotin-labeled DNA probes were detected by chemiluminescence using a LightShift Chemiluminescent EMSA Kit (Thermo Fisher Scientific). All experimental procedures were performed according to the manufacturer’s instructions, with the exception that the binding buffer condition was changed. Gel mobility shift assays were performed by incubating 10 fmol of the labeled probe with 30 pmol of proteins (MBP-SphR or MBP) in binding buffer (10 mM Tris-HCl, pH 7.5 containing 50 mM KCl, 1 mM dithiothreitol, 1 mM EDTA, 3 mM MgCl_2_, 50 μg/ml bovine serum albumin (BSA), 4% glycerol, 0.05% NP-40, and 50 ng/μl poly(dI-dC)) at 30 °C for 30 min. Five microliters of 5x Loading Buffer was added to the 20-μl reaction before being loaded onto a 5% native polyacrylamide gel in 0.5X TBE. After electrophoreses at 100 V for 70 min, samples were transferred onto a positively charged nylon membrane (Biodyne B, Pall Corporation, Port Washington, NY, USA) in 0.5x TBE at 380 mA for 30 min. Transferred DNAs were cross-linked to the membrane at 120 mJ/cm^2^ and detected using horseradish peroxidase-conjugated streptavidin according to the manufacturer’s instructions. The DNA band signal was visualized using Ez-Capture (ATTO CORPORATION, Tokyo, Japan).

### Biotin-Sph binding assay

Biotin-Sph (1 nmol) and MBP-SphR (0.2 nmol) were mixed in 100 μl of binding buffer (20 mM Tris-HCl, pH 7.5 containing 100 mM KCl, 2 mM DTT, 8% glycerol, 6 mM MgCl_2_, 100 μg/ml BSA, 2 mM EDTA, and 1% NP-40) and incubated at room temperature for 30 min. Dynabeads MyOne Streptavidin T1 (Thermo Fisher Scientific) was added to the mixture and incubated at room temperature for 30 min. Beads were separated using a magnet, the supernatant containing non-bound MBP-SphR was removed, and the beads were washed three times with 0.1% NP-40 in TBS using magnetic separation. The beads obtained were suspended in 45 μl of SDS sample buffer. After boiling at 100 °C for 5 min, the sample (20 μl) was subjected to SDS-PAGE and stained by EzStain Aqua (ATTO). The stained gel was scanned and band intensities were analyzed using Multi Gauge software version 3.1 (FUJIFILM Corporation, Tokyo, Japan).

## Additional Information

**How to cite this article**: Okino, N. and Ito, M. Molecular mechanism for sphingosine-induced *Pseudomonas* ceramidase expression through the transcriptional regulator SphR. *Sci. Rep.* **6**, 38797; doi: 10.1038/srep38797 (2016).

**Publisher's note:** Springer Nature remains neutral with regard to jurisdictional claims in published maps and institutional affiliations.

## Supplementary Material

Supplementary Information

## Figures and Tables

**Table 1:** Microarray results of increases in genes in *P. aeruginosa* PAO1 in the presence of SM.

PA number	Gene name	Gene description	Fold change*	P-value
PA5328	sphB	—	44.705	2.42E-07
PA5327	sphC	—	30.952	2.82E-06
PA0845	cerN	neutral ceramidase	18.986	4.91E-06
PA5326	sphD	—	18.219	1.63E-05
PA5325	sphA	—	6.835	0.0040980
PA0284	—	hypothetical protein	3.952	0.0002468
PA0283	sbp	sulfate-binding protein precursor	3.279	0.0075540
PA4443	cysD	ATP sulfurylase small subunit	3.183	0.0030508
PA0281	cysW	sulfate transport protein CysW	2.904	0.0030912
PA3450	—	probable antioxidant protein	2.864	0.0012612
PA2204	—	probable binding protein component of ABC transporter	2.702	0.0040892
PA4217	phzS	flavin-containing monooxygenase	2.623	0.0255484
PA4225	pchF	pyochelin synthetase	2.484	0.0472385
PA5471	armZ	—	2.340	0.0452318
PA4224	pchG	pyochelin biosynthetic protein PchG	2.335	0.0463765
PA1838	cysI	sulfite reductase	2.331	0.0097622
PA1984	exaC	NAD+ dependent aldehyde dehydrogenase ExaC	2.305	0.0134713
PA4442	cysN	ATP sulfurylase GTP-binding subunit/APS kinase	2.294	0.0304196
PA2426	pvdS	sigma factor PvdS	2.285	0.0049072
PA1837	—	hypothetical protein	2.271	0.0095659
PA4351	OlsA	—	2.238	0.0282751
PA0280	cysA	sulfate transport protein CysA	2.171	0.0079830
PA0843	plcR	phospholipase accessory protein PlcR precursor	2.074	0.0009660
PA0844	plcH	hemolytic phospholipase C precursor	2.073	0.0003768
PA3397	fpr	ferredoxin–NADP+ reductase	2.070	0.0014331
PA5117	typA	regulatory protein TypA	2.017	0.0016560
PA5373	betB	betaine aldehyde dehydrogenase	2.002	0.0062817
PA0654	speD	S-adenosylmethionine decarboxylase proenzyme	1.993	0.0081092
PA1325	—	conserved hypothetical protein	1.927	0.0364686
PA0201	—	hypothetical protein	1.910	0.0087476
PA4023	—	probable transport protein	1.888	0.0124535
PA4673	—	conserved hypothetical protein	1.826	0.0376535
PA3931	—	conserved hypothetical protein	1.824	0.0025698
PA5504	—	D-methionine ABC transporter membrane protein	1.810	0.0294901
PA1326	ilvA2	threonine dehydratase, biosynthetic	1.801	0.0351341
PA3655	tsf	elongation factor Ts	1.794	0.0130025
PA3820	secF	secretion protein SecF	1.775	0.0105703
PA3742	rplS	50S ribosomal protein L19	1.766	0.0322291
PA2405	—	hypothetical protein	1.755	0.0035630
PA0282	cysT	sulfate transport protein CysT	1.751	0.0318132
PA2970	rpmF	50S ribosomal protein L32	1.721	0.0178875
PA0730	—	probable transferase	1.721	0.0205574
PA4671	—	probable ribosomal protein L25	1.719	0.0398995
PA5528	—	hypothetical protein	1.701	0.0169662
PA5024	—	conserved hypothetical protein	1.688	0.0310829
PA1964	—	probable ATP-binding component of ABC transporter	1.687	0.0496123
PA4602	glyA3	serine hydroxymethyltransferase	1.684	0.0295932
PA2407	—	probable adhesion protein	1.678	0.0024510
PA3743	trmD	tRNA (guanine-N1)-methyltransferase	1.675	0.0315963
PA1800	tig	trigger factor	1.674	0.0112701
PA0904	lysC	aspartate kinase alpha and beta chain	1.668	0.0147283
PA4672	—	peptidyl-tRNA hydrolase	1.650	0.0427402
PA2786	—	hypothetical protein	1.643	0.0004732
PA5217	—	probable binding protein component of ABC iron transporter	1.641	0.0170144
PA2033	—	hypothetical protein	1.634	0.0410839
PA5374	betI	transcriptional regulator BetI	1.630	0.0179701
PA4588	gdhA	glutamate dehydrogenase	1.629	0.0278855
PA0672	hemO	heme oxygenase	1.624	0.0071025
PA2634	aceA	isocitrate lyase AceA	1.605	0.0295016
PA4273	rplA	50S ribosomal protein L1	1.603	0.0378134
PA0187	—	hypothetical protein	1.602	0.0019591
PA3446	—	conserved hypothetical protein	1.597	0.0080397
PA0277	—	conserved hypothetical protein	1.594	0.0476063
PA5316	rpmB	50S ribosomal protein L28	1.594	0.0217275
PA0662	argC	N-acetyl-gamma-glutamyl-phosphate reductase	1.591	0.0290015
PA2062	—	probable pyridoxal-phosphate dependent enzyme	1.587	0.0163330
PA5192	pckA	phosphoenolpyruvate carboxykinase	1.572	0.0085155
PA3745	rpsP	30S ribosomal protein S16	1.569	0.0033692
PA3744	rimM	16S rRNA processing protein	1.568	0.0079584
PA5425	purK	phosphoribosylaminoimidazole carboxylase	1.566	0.0264866
PA4854	purH	phosphoribosylaminoimidazolecarboxamide formyltransferase	1.566	0.0452696
PA4566	obg	GTP-binding protein Obg	1.565	0.0333140
PA3821	secD	secretion protein SecD	1.564	0.0070363
PA2404	—	hypothetical protein	1.561	0.0088869
PA2408	—	probable ATP-binding component of ABC transporter	1.550	0.0035579
PA4670	prs	ribose-phosphate pyrophosphokinase	1.548	0.0083203
PA0276	—	hypothetical protein	1.542	0.0247704
PA2427	—	hypothetical protein	1.534	0.0030403
PA2971	—	conserved hypothetical protein	1.532	0.0295900
PA4428	sspA	stringent starvation protein A	1.531	0.0283718
PA3313	—	hypothetical protein	1.530	0.0465274
PA4265	tufA	elongation factor Tu	1.529	0.0009703
PA4270	rpoB	DNA-directed RNA polymerase beta chain	1.529	0.0399673
PA3410	hasI	—	1.527	0.0368919
PA3014	faoA	fatty-acid oxidation complex alpha-subunit	1.522	0.0273633
PA4269	rpoC	DNA-directed RNA polymerase beta* chain	1.520	0.0414493
PA3763	purL	phosphoribosylformylglycinamidine synthase	1.517	0.0424190
PA4271	rplL	50S ribosomal protein L7/L12	1.515	0.0055188
PA5239	rho	transcription termination factor Rho	1.514	0.0441378
PA4745	nusA	N utilization substance protein A	1.513	0.0066330
PA5560	atpB	ATP synthase A chain	1.510	0.0213948
PA5347	—	hypothetical protein	1.507	0.0383823
PA4935	rpsF	30S ribosomal protein S6	1.506	0.0088829
PA2850	ohr	organic hydroperoxide resistance protein	1.505	0.0490325

Genes that are up-regulated by more than 1.5-fold with a p value of <0.05 in the presence of SM from the control were shown (n = 3).

^*^Fold change values were the average of three independent experiments.
