# Supplementary material for: Molecular mechanism for sphingosine-induced Pseudomonas ceramidase expression through the transcriptional regulator SphR
Source: Sci Rep. 2016 Dec 12;6:38797. doi: 10.1038/srep38797 (PMC5150637; doi:10.1038/srep38797)
Supplement: Supplementary Information [file srep38797-s1.pdf]

Molecular mechanism for sphingosine-induced *Pseudomonas* ceramidase expression through the transcriptional regulator SphR

Nozomu Okino[1,*] and Makoto Ito[1]

[1]The Department of Bioscience and Biotechnology, Graduate School of Bioresource and Bioenvironmental Sciences, Kyushu University, 6-10-1 Hakozaki, Higashi-ku, Fukuoka 812-8581, Japan

* Corresponding author: Nozomu Okino, Department of Bioscience and Biotechnology, Graduate School of Bioresource and Bioenvironmental Sciences, Kyushu University, 6-10-1 Hakozaki, Higashi-ku, Fukuoka 812-8581, Japan, E-mail: nokino@agr.kyushu-u.ac.jp

Supplementary Table S1

**Construction of the strain AN17-BGT**	
PaCD-5k-Infu1	CGACTCTAGAGGATCCCGGACAAACAGGCTCTGTCGCGGG
PaCD-5k-Infu2	CGGTACCCGGGGATCCTAGGCGTCCTCCTTGCGCGCGTCG
PaCD-5’UTR	GGCGACCCTCTTCTTGTTTTCCGCCGCTG
PaCD-3’UTR	CGGGCTCCAGCCAAGGTTGCGAGATTCG
PaCD-5’UTR-LacZ-5’	AAGAAGAGGGTCGCCATGACCATGATTACGGATTCACTGG
PaCD-3’UTR-LacZ-3’	CTTGGCTGGAGCCCGTTATTTTTGACACCAGACCAACTGG
PaCD3’UTR-3’U	CAAACATGAGAATTCCCGATAAATTATCGCCATGAAACCATCGGC
PaCD3’UTR-5’L	AAGAATTGGAGCCAACGATATGACCTTGAGCGACAACTTAGAAG
pBR322-U1-PaCD3’	GCGATAATTTATCGGGAATTCTCATGTTTGACAGCTTATC
pBR322-L1-PaCD3’	CTCAAGGTCATATCGTTGGCTCCAATTCTTGGAGTGG
PaCDPro-5k-Infu1	GCAGGTCGACGGATCCTAGGCGTCCTCCTTGCGCGCGTCG
PaCDPro-5k-Infu2	GAATTCCCGGGGATCCCGGACAAACAGGCTCTGTCGCGGG
**Construction of SphR mutant and complementation strains**	
SphR-1000-Infu1	CGACTCTAGAGGATCCCCGAAACCGAACACCCGTACGGTC
SphR-1000-Infu2	CGGTACCCGGGGATCCGGCAGTGGATTCCTCGCTGCCCGT
SphR-KO-InfuU1	CAAACATGAGAATTCTGGAGGAACCGCCATGGCGCGCCG
SphR-KO-InfuL1	AAGAATTGGAGCCAAGGGCGAAATCCTGAGCCGGTCTCG
pBR322U1new	GAATTCTCATGTTTGACAGCTTATC
pBR322L1331new	TTGGCTCCAATTCTTGGAGTGG
pMMB66HE-U1	AATTCAGCTTGGCTGTTTTGGCGG
pMMB66HE-L1	AATTCCCGGGGATCCGTCGACC
aacC4-1-Infu1	GGATCCCCGGGAATTCTGATAGTTTGGCTGTGAGCAA
aacC4-1-Infu2	CAGCCAAGCTGAATTCGCTC TCGGGTAACATCAAGG
SphR-U1-Infu1	GCAGGTCGACGGATCCCCAGAACAAGAACGAAGCCGAGAC
SphR-L1-Infu2	GAATTCCCGGGGATCCTGCTGGAAATCTTCACCGACAGCG
pMMB66HE-aacC4-U1	GATCCCCGGGAATTCTGATAGTTTG
pMMB66HE-aacC4-L1	GATCCGTCGACCTGCAGCCAAG
**Expression and purification of MBP-SphR**	
SphR-pMAL-Infu1	TGTCCATGGGCGGCCGCATGCACAACAATGCCGCCGATGCG
SphR-pMAL-Infu2	TACCTGCAGGGAATTCTTAGCGTCGCGCGGCCAGCGTG
**Electrophoretic Mobility Shift Assay**	
PaCDPro-200U	ACGCAGGCGGCCTTTTTTTACC

PaCDPro-81L	GGTTGCCACTGTAGTGCCAG
**5'-Race**	
PaCDProRT	TGGCGTTGAA GGTCT
PaCDPro-A1	AGGAGTAACCCATCATGC
PaCDProS1	GAAGTACCCC GGTGTCTA
PaCDPro-A2	GAATGCGGAA CGTGACAT
PaCDProS2	GACGAGAACA ACGTGATGCT
**Realtime PCR**	
PaCD-L1870	TAGTCGCTTTCGAGGCAGAT
PaCD-U1733	GCACCGAGAAGACTTTCCTG
PA3001-L629	TCTTGATGCCGTACTGGGTGTAGT
PA3001-U515	GCACCATCACCATCGACGAAGAAA
**Promoter analysis**	
PaCDPro2-Infu1	ATGCTCTTCGGATCCTCTTCGGCATCTGGATGA
PaCDPro2-Infu2-1	CGTCAGGATGGATCCGAATTCTCATGTTTGACAGCTTATC
pMMBdel-U1-BamHI	GGATCCATCCTGACGGATGGCCTTTTTGC
pMMBdel-L1-BamHI	GGATCCGAAGAGCATACATCTGGAAGCAAA G
PaCDPro-500-U1	TGGATGATCTGGCACTACAGTGGCAACCAG
PaCDPro-500-L1	AGTGCCAGATCATCCAGATGCCGAAGAGGATC
PaCDPro-400-U1	TGGATGATACGCAGGCGGCCTTTTTTTACCC
PaCDPro-400-L1	GCCTGCGTATCATCCAGATGCCGAAGAGGATC
PaCDPro0-U	TGGATGATAGGCGAAGCAGGCTCCGGCC
PaCDPro0-L	CTTCGCCTATCATCCAGATGCCGAAGAGGATCC
PaCDPro-200-U1	TGGATGATGCGAGAGGGCGCTGGTGAAATAA
PaCDPro-200-L1	CCTCTCGCATCATCCAGATGCCGAAGAGGATC
PaCDPro135-U	TGGATGATTTCCTGTCCGCTACGCCCCC
PaCDPro135-L	GACAGGAAATCATCCAGATGCCGAAGAGGATCC
PaCDPro155-U	TGGATGATGATGGCCGGCGCTCACCCC
PaCDPro155-L	CGGCCATCATCATCCAGATGCCGAAGAGGATCC
PaCDPro175-U	TGGATGATCTGCCTTGCCGTCCATGCAGG
PaCDPro175-L	CAAGGCAGATCATCCAGATGCCGAAGAGGATCC
PaCDPro3M1-U1	TGCAGGAAAGCCGGCGCTCACCCCCTT
PaCDPro3M1-L1	CGCCGGCTTTCCTGCATGGACGGCAAGG
PaCDPro3M2-U1	AGGATGGAAAGCGCTCACCCCCTTCCTG

PaCDPro3M2-L1	TGAGCGCTTTCCATCCTGCATGGACGGC
PaCDPro3M3-U1	CGGCGCTAACCCCCTTCCTGTCCGCT
PaCDPro3M3-L1	AGGGGGTTAGCGCCGGCCATCCTGCA
PaCDPro3M4-U1	GCGCTCAAAAACTTCCTGTCCGCTACGCC
PaCDPro3M4-L1	CAGGAAGTTTTTGAGCGCCGGCCATCCTG

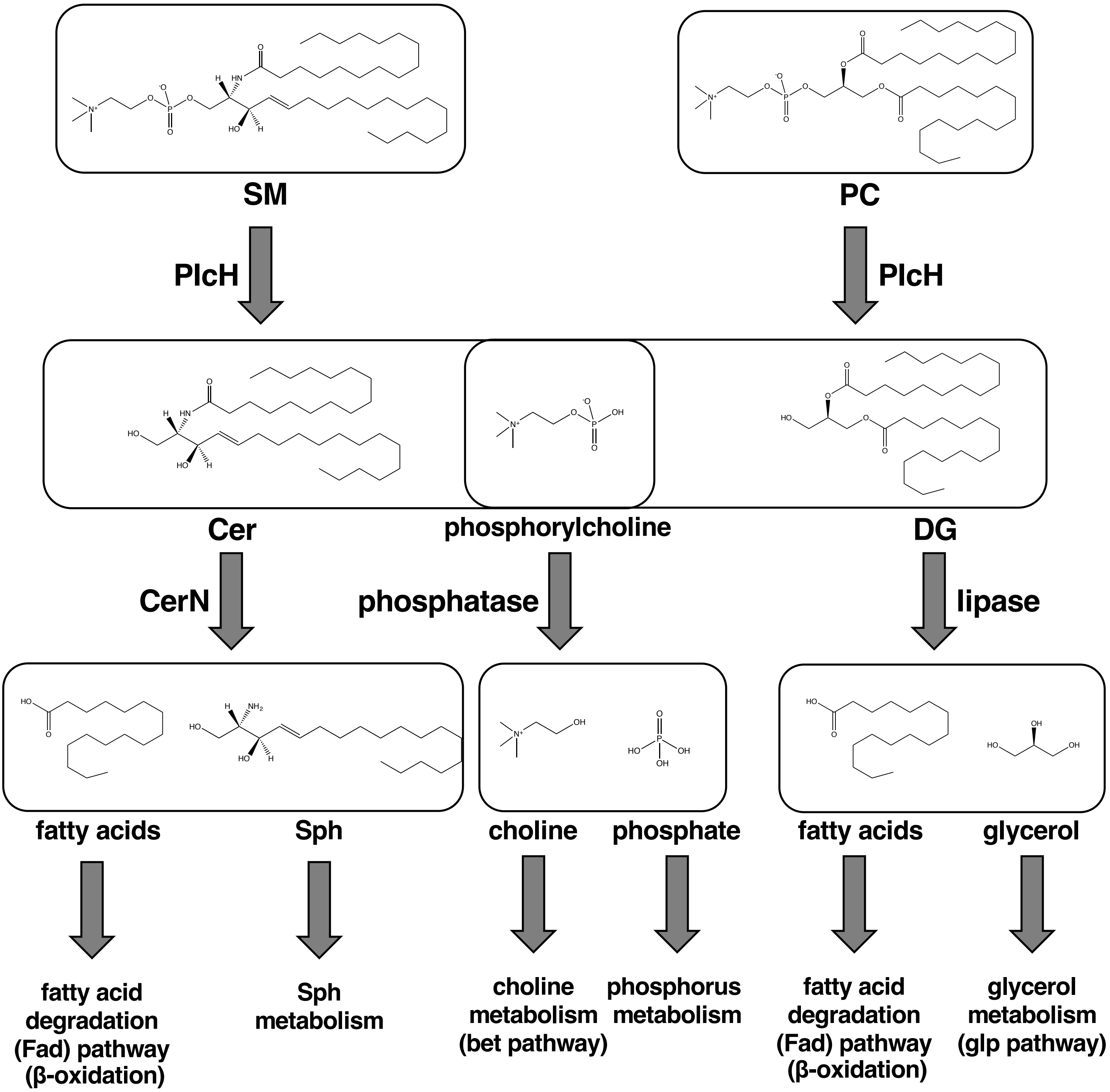

Supplementary Figure S1. Metabolic pathway for SM and PC in *P. aeruginosa*.
SM partly shares a metabolic pathway with PC; however, the metabolic pathways of Sph and glycerol are completely different from each other in *P. aeruginosa*. CerN; ceramidase, Cer; ceramide, PC; phosphatidylcholine, PlcH; hemolytic phospholipase C, SM; sphingomyelin, Sph, sphingosine.

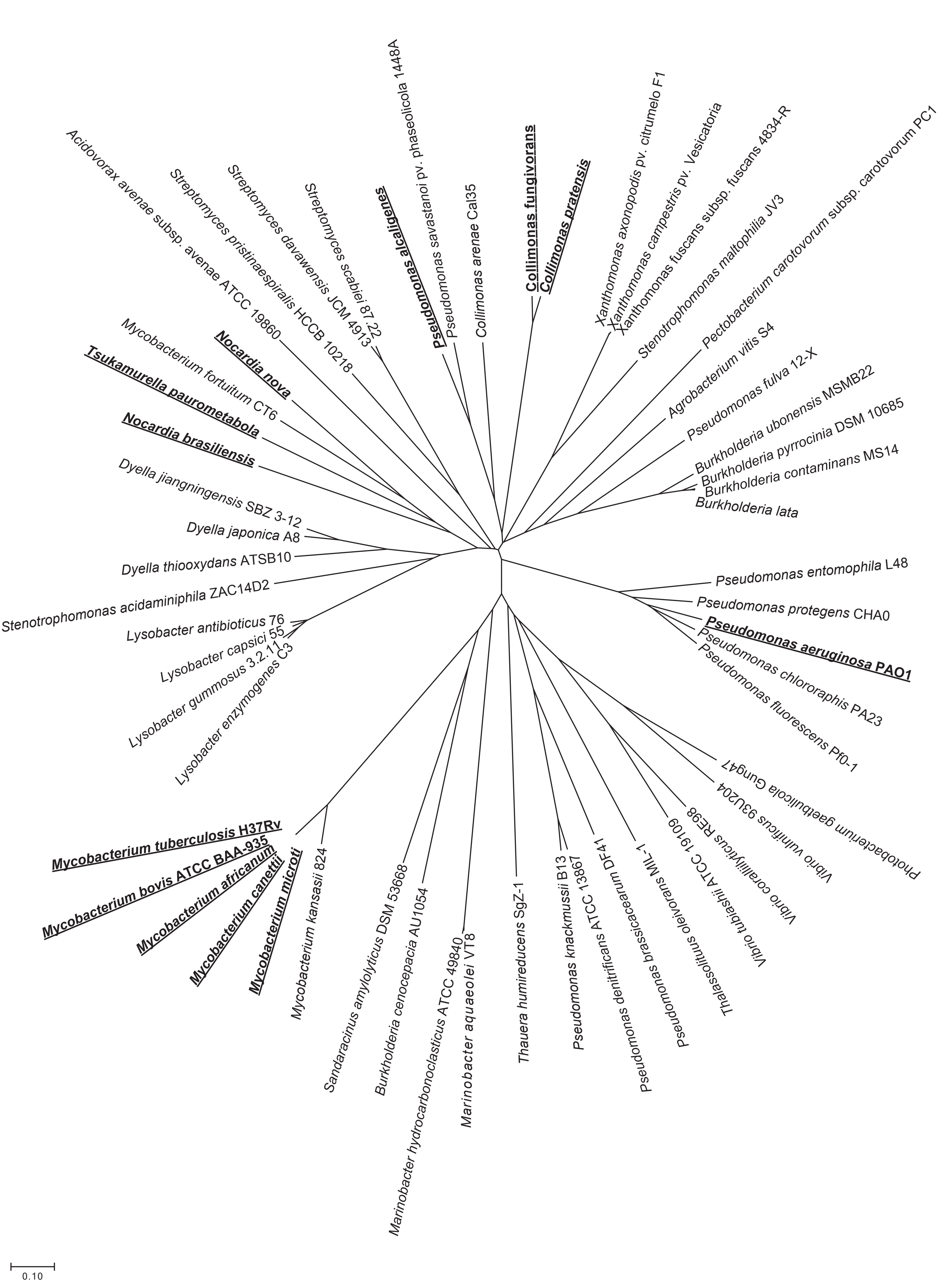

Supplementary Figure S2. Phylogenetic tree of SphR and SphR homologues.
Amino acid sequences of SphR and SphR homologues were analyzed using the neighbor-joining method with MEGA 7[1].
Bacteria possessing both SphR and CerN were indicated by bold letters with an underline.

Supplementary reference

1. Kumar, S., Stecher, G. & Tamura, K. MEGA7: Molecular Evolutionary Genetics Analysis Version 7.0 for Bigger Datasets. *Mol. Biol. Evol.* **33**, 1870-1874 (2016).
